# 4-[(*tert*-Butyl­diphenyl­sil­yloxy)meth­yl]pyridazin-3(2*H*)-one

**DOI:** 10.1107/S1600536813032212

**Published:** 2013-11-30

**Authors:** María Carmen Costas-Lago, Tamara Costas, Noemí Vila, Pedro Besada

**Affiliations:** a Department of Organic Chemistry, University of Vigo, E-36310 Vigo, Spain

## Abstract

In the title compound, C_21_H_24_N_2_O_2_Si, the carbonyl group of the heterocyclic ring and the O atom of the silyl ether group are placed toward opposite sides and the *tert*-butyl and pyridazinone moieties are *anti*-oriented across the Si—O bond [torsion angle = −168.44 (19)°]. In the crystal, mol­ecules are assembled into inversion dimers through co-operative N—H⋯O hydrogen bonds between the NH groups and O atoms of the pyridazinone rings of neighbouring mol­ecules. The dimers are linked by π–π inter­actions involving adjacent pyridazinone rings [centroid–centroid distance = 3.8095 (19) Å], generating ladder-like chains along the *b-*axis direction. The chains are further linked into a two-dimensional network parallel to the *ab* plane through weak C—H⋯π inter­actions.

## Related literature

For background to pyridazinone analogues displaying biological activities, see: Siddiqui *et al.* (2010 ▶); Costas *et al.* (2010 ▶); Abouzid & Bekhit (2008 ▶); Cesari *et al.* (2006 ▶); Rathish *et al.* (2009 ▶); Al-Tel (2010 ▶); Suree *et al.* (2009 ▶); Tao *et al.* (2011 ▶). For related structures, see: Costas *et al.* (2010 ▶); Costas-Lago *et al.* (2013 ▶).
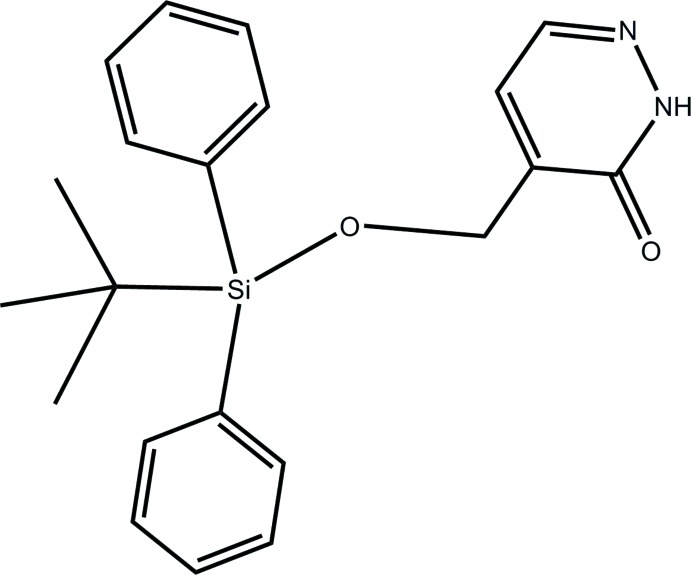


## Experimental

### 

#### Crystal data

C_21_H_24_N_2_O_2_Si
*M* *_r_* = 364.51
Monoclinic, 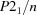

*a* = 10.774 (4) Å
*b* = 7.988 (3) Å
*c* = 24.681 (10) Å
β = 100.207 (7)°
*V* = 2090.5 (14) Å^3^
*Z* = 4
Mo *K*α radiation
μ = 0.13 mm^−1^
*T* = 293 K
0.48 × 0.41 × 0.23 mm

#### Data collection

Bruker SMART 1000 CCD diffractometer
Absorption correction: multi-scan (*SADABS*; Sheldrick, 1996 ▶) *T*_min_ = 0.707, *T*_max_ = 0.746
25187 measured reflections
5045 independent reflections
3076 reflections with *I* > 2σ(*I*)
*R*_int_ = 0.038

#### Refinement

*R*[*F* ^2^ > 2σ(*F* ^2^)] = 0.046
*wR*(*F* ^2^) = 0.148
*S* = 1.00
5045 reflections
242 parameters
H atoms treated by a mixture of independent and constrained refinement
Δρ_max_ = 0.30 e Å^−3^
Δρ_min_ = −0.24 e Å^−3^

### 

Data collection: *SMART* (Bruker, 1998 ▶); cell refinement: *SAINT* (Bruker, 1998 ▶); data reduction: *SAINT*; program(s) used to solve structure: *SIR2004* (Burla *et al.*, 2005 ▶); program(s) used to refine structure: *SHELXL97* (Sheldrick, 2008 ▶); molecular graphics: *PLATON* (Spek, 2003 ▶) and *Mercury* (Macrae *et al.*, 2006 ▶); software used to prepare material for publication: *SHELXTL* (Sheldrick, 2008 ▶).

## Supplementary Material

Crystal structure: contains datablock(s) I, New_Global_Publ_Block. DOI: 10.1107/S1600536813032212/lr2118sup1.cif

Structure factors: contains datablock(s) I. DOI: 10.1107/S1600536813032212/lr2118Isup2.hkl

Supplementary material file. DOI: 10.1107/S1600536813032212/lr2118Isup3.cml

Additional supplementary materials:  crystallographic information; 3D view; checkCIF report

## Figures and Tables

**Table 1:** Hydrogen-bond geometry (Å, °) *Cg*2 is the centroid of the C8′–C13′ ring

*D*—H⋯*A*	*D*—H	H⋯*A*	*D*⋯*A*	*D*—H⋯*A*
N2—H2⋯O3^i^	0.93 (3)	1.84 (3)	2.764 (2)	176 (2)
C6—H6⋯*Cg*2^ii^	0.93	2.76	3.637 (3)	138

Symmetry codes: (i) 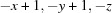; (ii) 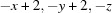.

**Fig. 1.**
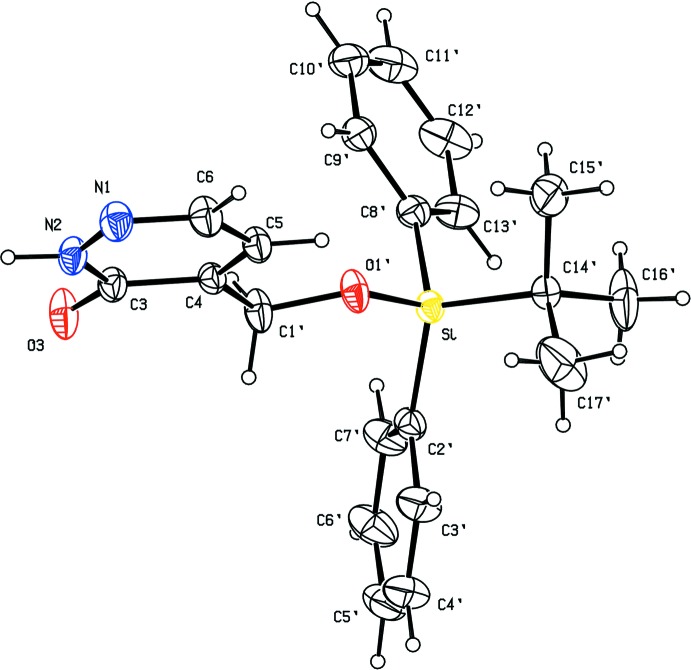
The molecular structure of (I) showing the atom-numbering scheme. Displacement ellipsoids are shown at the 20% probability level.

**Fig. 2.**
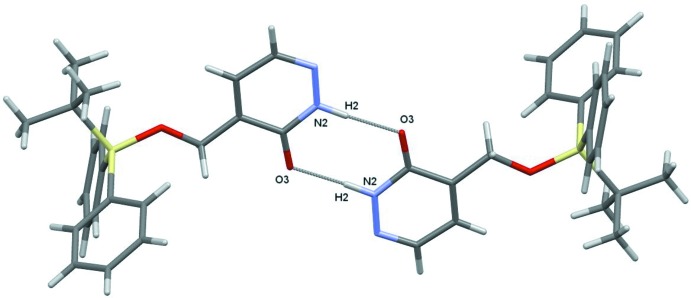
View of supramolecular dimer generated by NH···O hydrogen bonds.

**Fig. 3.**
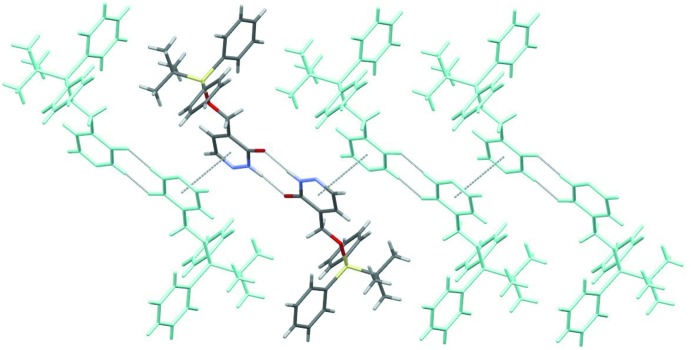
View of the ladder chain along crystallographic b axis generated by π–π interactions.

**Fig. 4.**
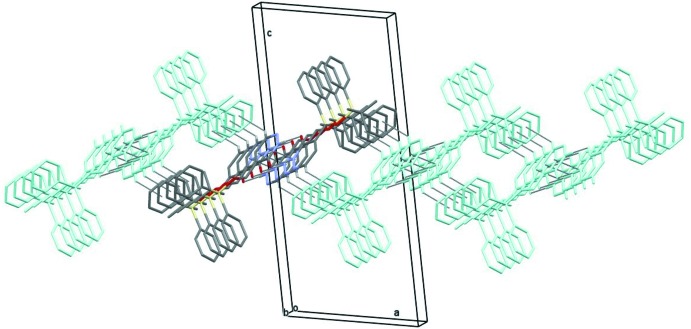
View of the two-dimensional organization generated by CH···π interactions (H atoms, no-involved in supramolecular structure, have been omitted to clarify).

## supplementary crystallographic information

### 1. Introduction

Pyridazin-3(2*H*)-one derivatives possess a wide range of biological activities, this fact together with the easy functionalization at various ring positions makes the pyridazinone nucleus a versatile pharmacophore to design and synthesize new drugs. For instance, an important number of pyridazinones have been reported as anti­hypertensive (Siddiqui *et al.*, 2010), anti­platelet (Costas *et al.*, 2010), anti-inflammatory (Abouzid & Bekhit, 2008), anti­nociceptive (Cesari *et al.*, 2006), anti­diabetic (Rathish *et al.*, 2009), anti­cancer (Al-Tel, 2010), anti­microbial (Suree *et al.*, 2009) or anti-histamine H_3_ agents (Tao *et al.*, 2011).

### 2.1. Synthesis and crystallization

A solution of 3-(*tert*-butyl­diphenyl­silyloxymethyl)-5-hy­droxy-5*H*-furan-2-ona (50 mg, 0.136 mmol) and hydrazine monohydrate (14 ml, 0.284 mmol) in ethanol (2 ml) was stirred at reflux for 4 h. The solvent was evaporated under reduced pressure and residue was purified by column chromatography on silica gel (hexane/ethyl acetate 4:1) to afford a colourless oil (16 mg, 32%). Single crystals suitable for X-ray analysis were grown from a chloro­form solution at room temperature.

### 2.2. Refinement

Crystal data, data collection and structure refinement details are summarized in Table 1. All H-atoms were positioned and refined using a riding model with d(C—H)= 0.93 Å, *U*_iso_ = 1.2*U*_eq_(C) for aromatic C—H groups,d(C—H)= 0.97 Å, *U*_iso_ = 1.2*U*_eq_(C) for CH_2_ group and d(C—H)= 0.96 Å, *U*_iso_ = 1.5*U*_eq_(C) for CH_3_ group; except for the hydrogen atoms of the NH group which were located from a Fourier-difference map and refined isotropically

### 3. Results and discussion

The compound I, an isomer of the 5-(*tert*-butyl­diphenyl­silyloxymethyl)­pyridazin-3(2*H*)-one (Costas-Lago *et al.*, 2013), was prepared in order to develop new pyridazinone analogues C4-substituted as anti­platelet agents. In the titled compound, the carbonyl group of the heterocyclic ring and the oxygen atom of the silyl ether group are placed toward opposite sides, this contrasts with the geometry found in the C5-substituted regioisomer and could explain the nearly flat disposition of the sequence C4—C1'-O1'-Si, with a torsion angle of -174.30 (15)°. The pyridazinone ring forms dihedral angles of 89.10 (8)° and 77.53 (7)°, respectively, with the C2'-C7' and C8'-C13' benzene rings, while the dihedral angle between both benzene rings is 48.41 (10)°.

The geometry of titled compound lets the assembly of molecules in supra­molecular organizations based on hydrogen bonding, π–π and CH···π inter­actions. The cooperative N—H···O hydrogen bonds between the NH group of one pyridazinone ring and the oxygen atom of an adjacent ring form supra­molecular dimers (Figure 2). These dimers are joined by π–π inter­actions involving also neighbouring pyridazinone rings [*Cg*(1): N1—N2—C3—C4—C5—C6; d[*Cg*(1)—*Cg*(1)^ii^]: 3.8095 (19) Å; d[*Cg*(1)···P(1)^ii^]: 3.4279 (8) Å; α: 0°; symmetry code ii: 1 - *x*, 2 - *y*, -*z*] resulting in a ladder chain along the crystallographic *b* axis (Figure 3). Finally, the linear chains are linked into a two-dimensional network through weak C—H···π inter­actions (Figure 4) involving CH groups of the pyridazinone rings and phenyl rings from neighbouring chains [C6—H6···*Cg*(2)^iii^; *Cg*(2): C8'-C9'-C10'-C11'-C12'-C13'; d[H···*Cg*(2)^iii^]: 2.890 Å; γ: 17.60°; symmetry code iii: 2 - *x*, -2 - *y*, -*z*]. In this case the pyridazinone ring arrangement prevents the three-dimensional growth observed in the C5-substituted regioisomer (Costas-Lago *et al.*, 2013).

### Crystal data

**Table:**

C_21_H_24_N_2_O_2_Si	*F*(000) = 776
*M**_r_* = 364.51	*D*_x_ = 1.158 Mg m^−^^3^
Monoclinic, *P*2_1_/*n*	Mo *K*α radiation, λ = 0.71073 Å
Hall symbol: -P 2yn	Cell parameters from 5037 reflections
*a* = 10.774 (4) Å	θ = 2.7–23.0°
*b* = 7.988 (3) Å	µ = 0.13 mm^−^^1^
*c* = 24.681 (10) Å	*T* = 293 K
β = 100.207 (7)°	Prism, colourless
*V* = 2090.5 (14) Å^3^	0.48 × 0.41 × 0.23 mm
*Z* = 4	

### Data collection

**Table:**

Bruker SMART 1000 CCD diffractometer	5045 independent reflections
Radiation source: fine-focus sealed tube	3076 reflections with *I* > 2σ(*I*)
Graphite monochromator	*R*_int_ = 0.038
φ and ω scans	θ_max_ = 28.0°, θ_min_ = 1.7°
Absorption correction: multi-scan (*SADABS*; Sheldrick, 1996)	*h* = −14→14
*T*_min_ = 0.707, *T*_max_ = 0.746	*k* = −10→10
25187 measured reflections	*l* = −32→32

### Refinement

**Table:**

Refinement on *F*^2^	Primary atom site location: structure-invariant direct methods
Least-squares matrix: full	Secondary atom site location: difference Fourier map
*R*[*F*^2^ > 2σ(*F*^2^)] = 0.046	Hydrogen site location: inferred from neighbouring sites
*wR*(*F*^2^) = 0.148	H atoms treated by a mixture of independent and constrained refinement
*S* = 1.00	*w* = 1/[σ^2^(*F*_o_^2^) + (0.0628*P*)^2^ + 0.7126*P*] where *P* = (*F*_o_^2^ + 2*F*_c_^2^)/3
5045 reflections	(Δ/σ)_max_ < 0.001
242 parameters	Δρ_max_ = 0.30 e Å^−^^3^
0 restraints	Δρ_min_ = −0.24 e Å^−^^3^

### Special details

**Table:**

Experimental. ^1^H-RMN (400 MHz, CDCl_3_) *δ* p.p.m.: 12.32 (s, 1H), 7.90 (d, 1H, *J*=4.0 Hz), 7.65 (m, 4H), 7.60 (m, 1H), 7.42 (m, 6H), 4.77 (d, 2H, *J*=1.7 Hz), 1.14 (s, 9H).
Geometry. All e.s.d.'s (except the e.s.d. in the dihedral angle between two l.s. planes) are estimated using the full covariance matrix. The cell e.s.d.'s are taken into account individually in the estimation of e.s.d.'s in distances, angles and torsion angles; correlations between e.s.d.'s in cell parameters are only used when they are defined by crystal symmetry. An approximate (isotropic) treatment of cell e.s.d.'s is used for estimating e.s.d.'s involving l.s. planes.
Refinement. Refinement of *F*^2^ against ALL reflections. The weighted *R*-factor *wR* and goodness of fit *S* are based on *F*^2^, conventional *R*-factors *R* are based on *F*, with *F* set to zero for negative *F*^2^. The threshold expression of *F*^2^ > σ(*F*^2^) is used only for calculating *R*-factors(gt) *etc*. and is not relevant to the choice of reflections for refinement. *R*-factors based on *F*^2^ are statistically about twice as large as those based on *F*, and *R*- factors based on ALL data will be even larger.

### Fractional atomic coordinates and isotropic or equivalent isotropic displacement parameters (Å^2^)

**Table:**

	*x*	*y*	*z*	*U*_iso_*/*U*_eq_	
Si	0.99974 (5)	0.99175 (7)	0.14301 (2)	0.04561 (17)	
N1	0.52674 (18)	0.8319 (2)	−0.06086 (7)	0.0653 (5)	
H2	0.487 (2)	0.611 (4)	−0.0346 (11)	0.086 (8)*	
N2	0.53846 (17)	0.7036 (2)	−0.02487 (7)	0.0574 (5)	
C3	0.62195 (19)	0.6898 (3)	0.02344 (8)	0.0526 (5)	
O3	0.62352 (16)	0.5633 (2)	0.05259 (7)	0.0788 (5)	
C4	0.70446 (17)	0.8303 (2)	0.03690 (8)	0.0467 (4)	
C5	0.6944 (2)	0.9595 (3)	0.00182 (8)	0.0560 (5)	
H5	0.7469	1.0521	0.0097	0.067*	
C6	0.6037 (2)	0.9546 (3)	−0.04720 (9)	0.0669 (6)	
H6	0.5993	1.0454	−0.0710	0.080*	
C1'	0.7958 (2)	0.8211 (3)	0.08991 (9)	0.0653 (6)	
H1'1	0.7505	0.8207	0.1206	0.078*	
H1'2	0.8444	0.7185	0.0913	0.078*	
O1'	0.87743 (14)	0.96100 (19)	0.09397 (6)	0.0627 (4)	
C2'	0.95347 (19)	0.9463 (3)	0.21122 (8)	0.0543 (5)	
C3'	0.8587 (3)	1.0372 (4)	0.22883 (12)	0.0840 (8)	
H3'	0.8172	1.1198	0.2059	0.101*	
C4'	0.8235 (3)	1.0095 (5)	0.27923 (15)	0.1019 (11)	
H4'	0.7594	1.0733	0.2897	0.122*	
C5'	0.8812 (3)	0.8910 (5)	0.31327 (12)	0.0971 (11)	
H5'	0.8589	0.8741	0.3476	0.117*	
C6'	0.9719 (3)	0.7968 (5)	0.29721 (11)	0.0973 (10)	
H6'	1.0109	0.7131	0.3203	0.117*	
C7'	1.0078 (2)	0.8232 (4)	0.24653 (10)	0.0762 (7)	
H7'	1.0702	0.7559	0.2363	0.091*	
C8'	1.1303 (2)	0.8497 (3)	0.13054 (8)	0.0552 (5)	
C9'	1.1229 (3)	0.7655 (3)	0.08041 (10)	0.0686 (6)	
H9'	1.0508	0.7771	0.0537	0.082*	
C10'	1.2208 (3)	0.6650 (3)	0.06949 (14)	0.0907 (9)	
H10'	1.2141	0.6115	0.0356	0.109*	
C11'	1.3262 (4)	0.6449 (4)	0.10820 (17)	0.1030 (11)	
H11'	1.3910	0.5763	0.1009	0.124*	
C12'	1.3376 (3)	0.7244 (4)	0.15767 (15)	0.0950 (10)	
H12'	1.4101	0.7105	0.1840	0.114*	
C13'	1.2403 (2)	0.8268 (3)	0.16862 (10)	0.0727 (7)	
H13'	1.2492	0.8813	0.2024	0.087*	
C14'	1.0445 (2)	1.2154 (3)	0.13265 (9)	0.0551 (5)	
C15'	1.0735 (3)	1.2322 (4)	0.07485 (11)	0.1004 (10)	
H15A	1.0925	1.3469	0.0681	0.151*	
H15B	1.0016	1.1971	0.0486	0.151*	
H15C	1.1446	1.1633	0.0714	0.151*	
C16'	1.1613 (3)	1.2610 (4)	0.17343 (14)	0.1250 (14)	
H16A	1.1790	1.3780	0.1702	0.188*	
H16B	1.2314	1.1963	0.1659	0.188*	
H16C	1.1477	1.2377	0.2101	0.188*	
C17'	0.9393 (4)	1.3371 (4)	0.1387 (2)	0.1496 (19)	
H17A	0.9230	1.3325	0.1756	0.224*	
H17B	0.8643	1.3072	0.1134	0.224*	
H17C	0.9643	1.4486	0.1308	0.224*	

### Atomic displacement parameters (Å^2^)

**Table:**

	*U*^11^	*U*^22^	*U*^33^	*U*^12^	*U*^13^	*U*^23^
Si	0.0432 (3)	0.0493 (3)	0.0416 (3)	−0.0068 (2)	0.0000 (2)	0.0014 (2)
N1	0.0713 (12)	0.0678 (12)	0.0512 (10)	−0.0148 (10)	−0.0044 (9)	0.0040 (9)
N2	0.0613 (11)	0.0557 (11)	0.0497 (10)	−0.0158 (9)	−0.0052 (8)	−0.0022 (8)
C3	0.0539 (12)	0.0536 (12)	0.0476 (11)	−0.0110 (9)	0.0012 (9)	−0.0008 (9)
O3	0.0872 (12)	0.0635 (10)	0.0720 (10)	−0.0326 (9)	−0.0229 (9)	0.0162 (8)
C4	0.0433 (10)	0.0514 (11)	0.0443 (10)	−0.0101 (8)	0.0048 (8)	−0.0012 (8)
C5	0.0542 (12)	0.0579 (12)	0.0537 (12)	−0.0164 (10)	0.0032 (9)	0.0023 (10)
C6	0.0732 (15)	0.0685 (15)	0.0541 (12)	−0.0161 (12)	−0.0021 (11)	0.0137 (11)
C1'	0.0649 (14)	0.0621 (14)	0.0607 (13)	−0.0267 (11)	−0.0119 (11)	0.0113 (11)
O1'	0.0576 (9)	0.0637 (9)	0.0584 (8)	−0.0250 (7)	−0.0126 (7)	0.0132 (7)
C2'	0.0464 (11)	0.0659 (13)	0.0495 (11)	−0.0099 (10)	0.0057 (9)	0.0012 (10)
C3'	0.0783 (17)	0.093 (2)	0.0888 (19)	0.0090 (15)	0.0360 (15)	0.0102 (15)
C4'	0.091 (2)	0.129 (3)	0.098 (2)	−0.010 (2)	0.0526 (19)	−0.014 (2)
C5'	0.0764 (19)	0.160 (3)	0.0581 (16)	−0.042 (2)	0.0211 (14)	−0.0041 (19)
C6'	0.0731 (17)	0.153 (3)	0.0643 (16)	−0.0122 (19)	0.0081 (14)	0.0392 (18)
C7'	0.0620 (14)	0.103 (2)	0.0648 (14)	0.0018 (14)	0.0150 (12)	0.0238 (14)
C8'	0.0649 (13)	0.0515 (12)	0.0507 (11)	0.0008 (10)	0.0140 (10)	0.0082 (9)
C9'	0.0959 (18)	0.0508 (13)	0.0650 (14)	−0.0103 (12)	0.0308 (13)	0.0029 (11)
C10'	0.140 (3)	0.0508 (14)	0.099 (2)	−0.0047 (17)	0.070 (2)	0.0005 (14)
C11'	0.126 (3)	0.0723 (19)	0.130 (3)	0.0320 (19)	0.077 (2)	0.034 (2)
C12'	0.0816 (19)	0.105 (2)	0.105 (2)	0.0336 (17)	0.0335 (17)	0.0447 (19)
C13'	0.0700 (15)	0.0858 (18)	0.0648 (14)	0.0173 (13)	0.0189 (12)	0.0162 (13)
C14'	0.0545 (12)	0.0520 (12)	0.0589 (12)	−0.0104 (10)	0.0100 (10)	−0.0043 (10)
C15'	0.164 (3)	0.0717 (18)	0.0710 (17)	−0.0341 (19)	0.0367 (19)	0.0069 (14)
C16'	0.146 (3)	0.107 (3)	0.103 (2)	−0.080 (2)	−0.030 (2)	0.0063 (19)
C17'	0.137 (3)	0.0609 (19)	0.278 (6)	0.0174 (19)	0.109 (4)	0.031 (3)

### Geometric parameters (Å, º)

**Table:**

Si—O1'	1.6420 (15)	C6'—C7'	1.390 (4)
Si—C2'	1.874 (2)	C6'—H6'	0.9300
Si—C8'	1.874 (2)	C7'—H7'	0.9300
Si—C14'	1.880 (2)	C8'—C13'	1.388 (3)
N1—C6	1.290 (3)	C8'—C9'	1.398 (3)
N1—N2	1.347 (3)	C9'—C10'	1.389 (4)
N2—C3	1.365 (3)	C9'—H9'	0.9300
N2—H2	0.93 (3)	C10'—C11'	1.358 (5)
C3—O3	1.239 (2)	C10'—H10'	0.9300
C3—C4	1.433 (3)	C11'—C12'	1.363 (5)
C4—C5	1.340 (3)	C11'—H11'	0.9300
C4—C1'	1.493 (3)	C12'—C13'	1.393 (4)
C5—C6	1.415 (3)	C12'—H12'	0.9300
C5—H5	0.9300	C13'—H13'	0.9300
C6—H6	0.9300	C14'—C16'	1.510 (3)
C1'—O1'	1.415 (2)	C14'—C15'	1.520 (3)
C1'—H1'1	0.9700	C14'—C17'	1.520 (4)
C1'—H1'2	0.9700	C15'—H15A	0.9600
C2'—C7'	1.374 (3)	C15'—H15B	0.9600
C2'—C3'	1.385 (3)	C15'—H15C	0.9600
C3'—C4'	1.381 (4)	C16'—H16A	0.9600
C3'—H3'	0.9300	C16'—H16B	0.9600
C4'—C5'	1.344 (5)	C16'—H16C	0.9600
C4'—H4'	0.9300	C17'—H17A	0.9600
C5'—C6'	1.346 (5)	C17'—H17B	0.9600
C5'—H5'	0.9300	C17'—H17C	0.9600
			
O1'—Si—C2'	109.06 (9)	C2'—C7'—H7'	119.2
O1'—Si—C8'	108.41 (10)	C6'—C7'—H7'	119.2
C2'—Si—C8'	110.90 (10)	C13'—C8'—C9'	116.3 (2)
O1'—Si—C14'	103.51 (9)	C13'—C8'—Si	122.96 (17)
C2'—Si—C14'	114.95 (10)	C9'—C8'—Si	120.66 (18)
C8'—Si—C14'	109.59 (10)	C10'—C9'—C8'	121.6 (3)
C6—N1—N2	115.14 (18)	C10'—C9'—H9'	119.2
N1—N2—C3	127.44 (18)	C8'—C9'—H9'	119.2
N1—N2—H2	116.9 (16)	C11'—C10'—C9'	120.1 (3)
C3—N2—H2	115.5 (16)	C11'—C10'—H10'	120.0
O3—C3—N2	120.83 (18)	C9'—C10'—H10'	120.0
O3—C3—C4	124.04 (18)	C10'—C11'—C12'	120.4 (3)
N2—C3—C4	115.12 (18)	C10'—C11'—H11'	119.8
C5—C4—C3	118.56 (18)	C12'—C11'—H11'	119.8
C5—C4—C1'	124.70 (18)	C11'—C12'—C13'	119.7 (3)
C3—C4—C1'	116.74 (17)	C11'—C12'—H12'	120.1
C4—C5—C6	119.70 (19)	C13'—C12'—H12'	120.1
C4—C5—H5	120.1	C8'—C13'—C12'	121.8 (3)
C6—C5—H5	120.1	C8'—C13'—H13'	119.1
N1—C6—C5	124.0 (2)	C12'—C13'—H13'	119.1
N1—C6—H6	118.0	C16'—C14'—C15'	108.6 (2)
C5—C6—H6	118.0	C16'—C14'—C17'	109.2 (3)
O1'—C1'—C4	109.16 (16)	C15'—C14'—C17'	108.4 (3)
O1'—C1'—H1'1	109.8	C16'—C14'—Si	110.01 (18)
C4—C1'—H1'1	109.8	C15'—C14'—Si	108.18 (16)
O1'—C1'—H1'2	109.8	C17'—C14'—Si	112.41 (17)
C4—C1'—H1'2	109.8	C14'—C15'—H15A	109.5
H1'1—C1'—H1'2	108.3	C14'—C15'—H15B	109.5
C1'—O1'—Si	125.32 (13)	H15A—C15'—H15B	109.5
C7'—C2'—C3'	115.6 (2)	C14'—C15'—H15C	109.5
C7'—C2'—Si	123.85 (18)	H15A—C15'—H15C	109.5
C3'—C2'—Si	120.58 (18)	H15B—C15'—H15C	109.5
C4'—C3'—C2'	122.3 (3)	C14'—C16'—H16A	109.5
C4'—C3'—H3'	118.8	C14'—C16'—H16B	109.5
C2'—C3'—H3'	118.8	H16A—C16'—H16B	109.5
C5'—C4'—C3'	120.3 (3)	C14'—C16'—H16C	109.5
C5'—C4'—H4'	119.8	H16A—C16'—H16C	109.5
C3'—C4'—H4'	119.8	H16B—C16'—H16C	109.5
C4'—C5'—C6'	119.3 (3)	C14'—C17'—H17A	109.5
C4'—C5'—H5'	120.3	C14'—C17'—H17B	109.5
C6'—C5'—H5'	120.3	H17A—C17'—H17B	109.5
C5'—C6'—C7'	120.8 (3)	C14'—C17'—H17C	109.5
C5'—C6'—H6'	119.6	H17A—C17'—H17C	109.5
C7'—C6'—H6'	119.6	H17B—C17'—H17C	109.5
C2'—C7'—C6'	121.6 (3)		
			
C6—N1—N2—C3	−0.3 (3)	C4'—C5'—C6'—C7'	1.3 (5)
N1—N2—C3—O3	−179.2 (2)	C3'—C2'—C7'—C6'	−2.0 (4)
N1—N2—C3—C4	0.9 (3)	Si—C2'—C7'—C6'	178.6 (2)
O3—C3—C4—C5	179.5 (2)	C5'—C6'—C7'—C2'	0.5 (5)
N2—C3—C4—C5	−0.7 (3)	O1'—Si—C8'—C13'	−170.38 (18)
O3—C3—C4—C1'	−0.8 (3)	C2'—Si—C8'—C13'	−50.7 (2)
N2—C3—C4—C1'	179.02 (19)	C14'—Si—C8'—C13'	77.3 (2)
C3—C4—C5—C6	0.0 (3)	O1'—Si—C8'—C9'	12.29 (19)
C1'—C4—C5—C6	−179.7 (2)	C2'—Si—C8'—C9'	132.00 (17)
N2—N1—C6—C5	−0.5 (4)	C14'—Si—C8'—C9'	−100.04 (18)
C4—C5—C6—N1	0.7 (4)	C13'—C8'—C9'—C10'	−0.1 (3)
C5—C4—C1'—O1'	−6.1 (3)	Si—C8'—C9'—C10'	177.36 (17)
C3—C4—C1'—O1'	174.22 (19)	C8'—C9'—C10'—C11'	0.8 (4)
C4—C1'—O1'—Si	−174.30 (15)	C9'—C10'—C11'—C12'	−0.8 (4)
C2'—Si—O1'—C1'	−45.6 (2)	C10'—C11'—C12'—C13'	0.2 (5)
C8'—Si—O1'—C1'	75.2 (2)	C9'—C8'—C13'—C12'	−0.5 (3)
C14'—Si—O1'—C1'	−168.44 (19)	Si—C8'—C13'—C12'	−178.0 (2)
O1'—Si—C2'—C7'	118.5 (2)	C11'—C12'—C13'—C8'	0.5 (4)
C8'—Si—C2'—C7'	−0.8 (2)	O1'—Si—C14'—C16'	−177.2 (2)
C14'—Si—C2'—C7'	−125.8 (2)	C2'—Si—C14'—C16'	64.0 (2)
O1'—Si—C2'—C3'	−60.8 (2)	C8'—Si—C14'—C16'	−61.7 (2)
C8'—Si—C2'—C3'	179.9 (2)	O1'—Si—C14'—C15'	−58.7 (2)
C14'—Si—C2'—C3'	54.9 (2)	C2'—Si—C14'—C15'	−177.52 (18)
C7'—C2'—C3'—C4'	1.8 (4)	C8'—Si—C14'—C15'	56.8 (2)
Si—C2'—C3'—C4'	−178.8 (2)	O1'—Si—C14'—C17'	61.0 (3)
C2'—C3'—C4'—C5'	−0.1 (5)	C2'—Si—C14'—C17'	−57.9 (3)
C3'—C4'—C5'—C6'	−1.5 (5)	C8'—Si—C14'—C17'	176.5 (3)

### Hydrogen-bond geometry (Å, º)

Cg2 is the centroid of the C8'–C13' ring

**Table:**

*D*—H···*A*	*D*—H	H···*A*	*D*···*A*	*D*—H···*A*
N2—H2···O3^i^	0.93 (3)	1.84 (3)	2.764 (2)	176 (2)
C6—H6···*Cg*2^ii^	0.93	2.76	3.637 (3)	138

Symmetry codes: (i) −*x*+1, −*y*+1, −*z*; (ii) −*x*+2, −*y*+2, −*z*.

## References

[bb1] Abouzid, K. & Bekhit, S. A. (2008). *Bioorg. Med. Chem.* **16**, 5547–5556. 10.1016/j.bmc.2008.04.007 18430576

[bb2] Al-Tel, T. H. (2010). *Eur. J. Med. Chem.* **45**, 5724–5731. 10.1016/j.ejmech.2010.09.029 20884086

[bb3] Bruker (1998). *SMART* and *SAINT* Bruker AXS Inc., Madinson, Wisconsin, USA.

[bb4] Burla, M. C., Caliandro, R., Camalli, M., Carrozzini, B., Cascarano, G. L., De Caro, L., Giacovazzo, C., Polidori, G. & Spagna, R. (2005). *J. Appl. Cryst.* **38**, 381–388.

[bb5] Cesari, N., Biancanali, C., Vergelli, C., Dal Piaz, V., Graziano, A., Biagini, P., Chelardini, C., Galeotti, N. & Giovannoni, P. (2006). *J. Med. Chem.* **49**, 7826–7835. 10.1021/jm060743g 17181165

[bb6] Costas, T., Besada, P., Piras, A., Acevedo, L., Yañez, M., Orallo, F., Laguna, R. & Terán, C. (2010). *Bioorg. Med. Chem. Lett.* **20**, 6624–6627. 10.1016/j.bmcl.2010.09.031 20880705

[bb7] Costas-Lago, M. C., Costas, T., Vila, N. & Terán, C. (2013). *Acta Cryst.* E**69**, o1826–o1827. 10.1107/S160053681303167X PMC3885075 24454251

[bb8] Macrae, C. F., Edgington, P. R., McCabe, P., Pidcock, E., Shields, G. P., Taylor, R., Towler, M. & van de Streek, J. (2006). *J. Appl. Cryst.* **39**, 453–457.

[bb9] Rathish, I. G., Javed, K., Bano, S., Ahmad, S., Alam, M. S. & Pillai, K. K. (2009). *Eur. J. Med. Chem.* **44**, 2673–2678. 10.1016/j.ejmech.2008.12.013 19171410

[bb10] Sheldrick, G. M. (1996). *SADABS.* University of Göttingen, Germany.

[bb11] Sheldrick, G. M. (2008). *Acta Cryst.* A**64**, 112–122. 10.1107/S0108767307043930 18156677

[bb12] Siddiqui, A. A., Mishra, R. & Shaharyar, M. (2010). *Eur. J. Med. Chem.* **45**, 2283–2290. 10.1016/j.ejmech.2010.02.003 20189270

[bb13] Spek, A. L. (2003). *J. Appl. Cryst.* **36**, 7–13.

[bb14] Suree, N., Yi, S. W., Thieu, W., Marohn, M., Damoiseaux, R., Chan, A., Jung, M. E. & Club, R. T. (2009). *Bioorg. Med. Chem.* **17**, 7174–7185. 10.1016/j.bmc.2009.08.067 PMC2888031 19781950

[bb15] Tao, M., Raddatz, R., Aimone, L. D. & Hudkins, R. L. (2011). *Bioorg. Med. Chem. Lett.* **21**, 6126–6130. 10.1016/j.bmcl.2011.08.045 21906941

